# Ketogenic Diet Improves Motor Performance but Not Cognition in Two Mouse Models of Alzheimer’s Pathology

**DOI:** 10.1371/journal.pone.0075713

**Published:** 2013-09-12

**Authors:** Milene L. Brownlow, Leif Benner, Dominic D’Agostino, Marcia N. Gordon, Dave Morgan

**Affiliations:** 1 Department of Molecular Pharmacology and Physiology, University of South Florida College of Medicine, Tampa, Florida, United States of America; 2 USF Health Byrd Alzheimer’s Institute, Tampa, Florida, United States of America; University of Florida, United States of America

## Abstract

Dietary manipulations are increasingly viewed as possible approaches to treating neurodegenerative diseases. Previous studies suggest that Alzheimer’s disease (AD) patients present an energy imbalance with brain hypometabolism and mitochondrial deficits. Ketogenic diets (KDs), widely investigated in the treatment and prevention of seizures, have been suggested to bypass metabolic deficits present in AD brain by providing ketone bodies as an alternative fuel to neurons. We investigated the effects of a ketogenic diet in two transgenic mouse lines. Five months old APP/PS1 (a model of amyloid deposition) and Tg4510 (a model of tau deposition) mice were offered either a ketogenic or a control (NIH-31) diet for 3 months. Body weight and food intake were monitored throughout the experiment, and blood was collected at 4 weeks and 4 months for ketone and glucose assessments. Both lines of transgenic mice weighed less than nontransgenic mice, yet, surprisingly, had elevated food intake. The ketogenic diet did not affect these differences in body weight or food consumption. Behavioral testing during the last two weeks of treatment found that mice offered KD performed significantly better on the rotarod compared to mice on the control diet independent of genotype. In the open field test, both transgenic mouse lines presented increased locomotor activity compared to nontransgenic, age-matched controls, and this effect was not influenced by KD. The radial arm water maze identified learning deficits in both transgenic lines with no significant differences between diets. Tissue measures of amyloid, tau, astroglial and microglial markers in transgenic lines showed no differences between animals fed the control or the ketogenic diet. These data suggest that ketogenic diets may play an important role in enhancing motor performance in mice, but have minimal impact on the phenotype of murine models of amyloid or tau deposition.

## Introduction

Alzheimer’s Disease (AD) is a chronic disorder that affects as many as 5.4 million people in the US and its incidence is expected to double by 2050 (Alzheimer’s Association, 2012). AD can affect people in different ways but the earliest and most prominent symptom is the progressive decline in memory. Pathologically, it is characterized by extracellular deposits of amyloid beta (Aβ), intracellular accumulation of neurofibrillary tangles, and neuronal loss. Several transgenic mouse models that express mutant genes demonstrated to cause dementia in humans replicate some of the histopathological and cognitive features of the disease. Some of these models express genes associated with familial Alzheimer’s disease (FAD), while others cause frontotemporal lobe dementia (FTD).

Other consistent features of dementias are hypometabolism in several brain areas but particularly in the hippocampus of AD patients [1,2] and frontal lobes of FTD patients [3,4], and impaired mitochondrial function [5]. Decreased cerebral glucose utilization is an early event in AD pathology and may precede the neuropathological deposition of Aβ and/or clinical symptoms [6] [7] by decades. Similarly, mitochondrial deficits precede the deposition of amyloid and tau in the brains of a triple transgenic mouse model of AD (3xTgAD).

Furthermore, a shift in brain metabolism has been reported in mouse models of AD, changing from utilization of glucose to increased ketogenesis during aging [8,9]. Ketone bodies (KBs) are a by-product of the breakdown of fat in the liver. The main endogenous KBs are β-hydroxybutyrate (BHB), acetoacetate and acetone. When KBs accumulate in the bloodstream, they cause a metabolic state called ketosis. Ketosis is a survival mechanism activated during prolonged fasting, starvation or lack of carbohydrate ingestion. During prolonged ketosis, the brain is capable of metabolizing ketone bodies as an alternate fuel, thus reducing its requirement for glucose [10]. Diets that are rich in fat and low in carbohydrate, known as ketogenic diets, mimic the effects of fasting and the lack of glucose/insulin signaling promotes a metabolic shift toward fatty acids utilization [11]. Blood ketone levels are typically modest, and well below those caused by the metabolic ketosis accompanying diabetes.

Ketogenic diets have been successfully used in the treatment and prevention of seizures in epilepsy [12-16]. The improved metabolic efficiency observed suggests that, besides its anticonvulsant properties, KDs may be useful for treating several other neurological disorders. Current models of neurodegenerative diseases showed positive outcomes induced by ketogenic diet such as increased motor neuron number in ALS transgenic models [17,18], reduced lesion volume after traumatic brain injury [19], increased cell survival and decreased seizure frequency in kainate-induced seizure models [20] and suppressed inflammatory cytokines and chemokines in an experimental model of multiple sclerosis [21].

Medium chain triglycerides are fatty acids that are rapidly metabolized in the liver to produce a mild state of ketosis [22,23]. Recent clinical trials in patients with AD or mild cognitive impairment have shown that an orally administered mixture of medium chain triglycerides (AC-1202, Accera, Inc.) can improve memory and attention in these individuals, and better performance was associated with higher β-hydroxybutyrate plasma levels. Cognitive facilitation was greater in ApoE4-negative adults with memory disorders [24].

Consequently, we investigated the effects of a diet rich in medium chain triglycerides and low in carbohydrate in two well-established mouse models with Alzheimer-like pathology, amyloid-depositing mice transgenic for mutant amyloid precursor protein and presenilin-1(APP+PS1) and tau-depositing mice transgenic for tau (Tg4510). We used these two mouse models to dissociate dietary effects on the deposition of amyloid and tau proteins separately because our recent evidence suggests that these two main pathologies may be regulated differently [25,26]. We demonstrated that the ketogenic diet effectively elevated circulating ketone bodies while reducing glucose levels. We examined effects of this diet on behavioral and histopathological outcomes in transgenic models with Alzheimer-like pathology and age-matched nontransgenic littermates.

Because AD requires many years to fully manifest in patients, an effective diet treatment could delay the rate of symptom progression and delay or prevent institutionalization. Therefore, we sought to study the effects of the KD in two well-established mouse models of Alzheimer’s pathology, dissociating its effects on the deposition of amyloid and tau proteins. To our knowledge, this is the first report of the effects of a ketogenic diet on a mouse model of tauopathy in the absence of amyloid.

## Materials and Methods

### Ethics Statement

All animal testing procedures were approved by the Institutional Animal Care and Use Committee of the University of South Florida and followed NIH guidelines for the care and use of laboratory animals (Approval ID number: 0054R).

### Ketogenic Diet

The ketogenic diet was devised by D. D’Agostino in consultation with a nutritionist at Teklad (Madison, WI). Briefly, the goal was to obtain a low carbohydrate, medium chain triglyceride-rich diet which induced ketosis, but did so without introducing high amounts of omega-6 or hydrogenated fats. We also wished to develop a diet which did not lead to weight loss, to avoid confounds of dietary or caloric restriction effects as a possible cause for any observed outcomes. A detailed list of macronutrient components of the diet used in this experiment is presented in Table 1.

**Table 1:** Diet Composition.

	NIH-31	Ketogenic Diet
	grams/kg	grams/kg
Casein	210	300
L-Cystine	3	2.86
Sucrose	200	0
Maltodextrin	100	0
Corn Starch	369	0
Cellulose (Fiber)	40	245.31
MCT Oil (Medium Chain Triglycerides)	0	270
Flaxseed Oil	21	70
Canola Oil	19	60
Mineral Mix Ca-P Deficient (79055)	13.4	18.5
Calcium Phosphate Dibasic CaHPO4	7	8.5
Calcium Carbonate CaCO3	7.3	10.75
40060 VM, Teklad	10	14
Ethoxyquin (Liquid)	0.1	0.08
**TOTAL**	**1000**	**1000**
Protein, % of kcal	23.8	22.4
Carbohydrate, % of kcal	62.2	0.5
Fat, % by kcal	14	77.1
Vitamin mix, % of kcal	1.3	1.2
kcal/g	3.0	4.7

### Mice

APP+PS1 mice [27] were acquired from our breeding colonies at the University of South Florida. These mice develop congophilic amyloid deposits by 3 months of age, with neuritic and glial involvement, which increase in number with aging, along with deficits in learning and memory [28]. The Tg4510 mouse carries a regulatable tau transgene with the fronto-temporal dementia associated mutation P301L mutation. Mutant tau expression is restricted largely to forebrain neurons by CaM kinase II-driven expression of the tet trans activator. These mice develop progressive pathology with the first discernible abnormally phosphorylated tau observed at 3 months of age, leading to readily detectable neuron loss by 6 months of age [29,30]. In this experiment, mice were given food and water *ad libitum* and maintained on a twelve-hour light/dark cycle and standard vivarium conditions. In order to assess the effects of a KD on mice with some pre-existing pathology, all animals were 5 months old at the start of the experiment. Animals were housed and treated according to institutional and National Institutes of Health standards. Nontransgenic mice used as a positive control for behavioral studies were littermates from the breeding of Tg4510 mice and are FVB/129S. APP+PS1 mice are on a mixed background of primarily C57/BL6 with smaller contributions from DBA/2, SW, SGL lines with the *rd1* mutation selected out of the breeding colony.

### Experimental Procedure

**Figure 1:**
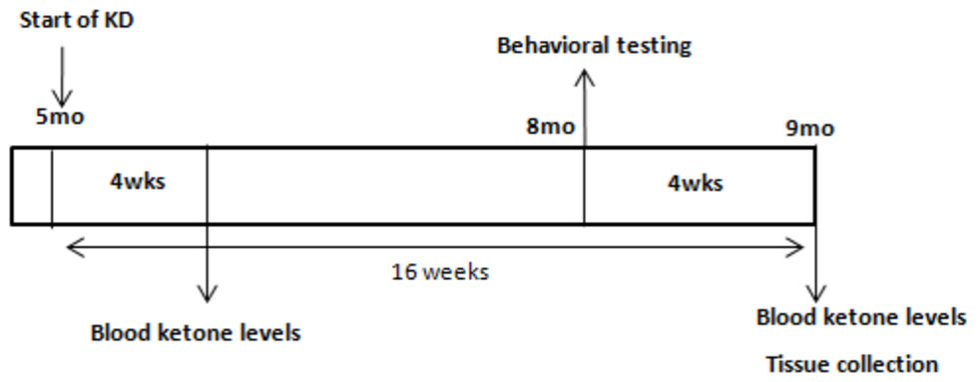
Experimental design for the study of a ketogenic diet using two mouse models of Alzheimer’s pathology. APP+PS1, Tg4510 and nontransgenic littermates received either a control (NIH-31) or a low carbohydrate, medium-chain triglyceride rich, ketogenic diet (KD) for 16 weeks. Blood was collected at 4 weeks and 16 weeks for measurement of circulating ketone and glucose levels. Near the end of the administration period, mice received a variety of behavioral tests. Tissue was collected after a 16 week diet administration.

Figure 1 depicts the experimental design and time course adopted in this experiment. Mice were randomly assigned to receive either ketogenic or NIH-31 control diet (n=10/group, *ad libitum*). Food was replaced three times and food consumption and body weight were monitored. The week before the commencement of the experiment, mice were transitioned to the new diet for a week by having gradually increasing quantities of KD offered in addition to their standard diet. Four weeks after the start of the diets, peripheral blood was collected, and non-fasting levels of β-hydroxybutyrate (BHB) and glucose were measured using a commercially available glucose/ketone meter (Nova Max Plus, Waltham, MA). After three months on their respective diets, the mice were submitted to a battery of behavioral testing. At the end of testing, mice were weighed, euthanized with a solution containing pentobarbital and transcardially perfused with 25 ml of 0.9% normal saline solution. Brains were collected immediately following perfusion. One hemisphere was dissected and immediately frozen on dry ice for biochemical analysis. The second hemisphere was immersion fixed in 4% phosphate-buffered paraformaldehyde for 24h. The fixed hemispheres were cryoprotected in successive incubations of 10%, 20% and 30% solutions of sucrose for 24h each. Subsequently, brains were frozen on a cold stage and sectioned in the horizontal plane (25 μm thickness) on a sliding microtome and stored in Dulbecco’s phosphate buffered saline (DPBS) with 10 mM sodium azide solution at 4°C. Every 8^th^ section was cut at 50 μm thickness for stereological counts of neurons and measurement of hippocampal volume in Nissl-stained sections.

### Behavioral Testing

The open field was used as a standard test of general activity. Animals were monitored for 15 minutes in a 40 cm square open field with a video tracking software, under moderate lighting. General activity levels were evaluated by measurements of horizontal and vertical activity.

Each animal was placed in a walled Y-maze for a single 5 minute trial. The sequence of arm entries and total number of arm choices were recorded. Spontaneous alternation (entering all three arms sequentially without repetition) was expressed as a percentage, as calculated according to the method of [31].

Motor performance was evaluated by an accelerating rotarod apparatus with a 3cm diameter rod starting at an initial rotation of 4 RPM slowly accelerating to 40 RPM over 5 minutes. Mice were expected to walk at the speed of rod rotation to keep from falling. The time spent on the rod during each of four trials per day for two consecutive days was measured. Testing was completed when the mouse fell off the rod (distance of 12 cm) onto a spring-cushioned lever, and rarely attained the maximal time allotted. Motor performance in an endurance version of the rotarod was assessed in a single trial on the third day. For this trial, the rod was accelerated to a lower maximum speed of 25 RPM and held constant, and the mice were allowed to stay on the rod for a maximum trial length of 1000s (the longest duration allowed by the software).

### Radial Arm Water Maze (RAWM)

A detailed description of this test has been previously published, complete with goal arm assignments and scoring sheets [32]. Briefly, the radial arm water maze contains 6 swim paths (arms) radiating out of an open central area with a hidden escape platform located at the end of one of the arms. The pool is surrounded by several extra-maze cues to allow spatial navigation. On each trial, the mouse was allowed to swim for up to 60 seconds to find the escape platform. The platform was located in the same arm on each trial. On day one mice were given 15 trials alternating between a visible platform (above the water) and a hidden platform (below the water). On day two, mice were given 15 additional trials with all the trials using a hidden platform. The start arm was varied for each trial so that mice relied upon spatial cues to solve the task instead of learning motor rules (i.e. second arm on the right). The goal arm for each mouse was different to avoid odor cues revealing the goal arm. Entry into an incorrect arm (all four limbs within the arm) was scored as an error. Failure to make an arm entry within 15 seconds was also scored as an error. The errors for blocks of 3 consecutive trials were averaged for data analysis. Mice averaging of 1 error or less by the end of day two are considered to have reached the learning criterion. On the third day, a reversal trial was performed with the goal platform placed in the arm 180° from the original location. Mice were given 15 trials all with a hidden platform. The open pool with a visible platform test was performed on the day following the reversal trial to confirm that all mice were capable of seeing and ascending the platform. The visible platform was elevated above the water surface and had an attached flag. For the open pool test, all visual cues were removed from the room so the mice relied only on their sight to find the platform. Latency to find and ascend the platform was recorded (60 seconds maximum).

### Fear Conditioning

Fear conditioning was used to assess memory formation that is especially sensitive to proper hippocampal function. For these experiments, an aversive stimulus (in this case a mild foot shock, 0.5mA) was paired with an auditory conditioned stimulus (white noise) within a novel environment. Freezing on the training day in response to the foot shock was used as an estimate of learning during the acquisition trial. Animals were placed in the fear conditioning apparatus for 3 min, then a 30 s acoustic conditioned stimulus (white noise, 70 dB) was delivered with a 0.5-mA shock (unconditioned stimulus) applied to the floor grid during the last 2 s of the CS. Training consisted of two mild shocks paired with two conditioned stimuli with a 2-min interval between each shock. For contextual memory, the mice were placed in the chamber and monitored for freezing to the context approximately 24hour after training (no shocks or auditory cue given) and tested for 3 min. Learning was assessed by measuring freezing behavior (i.e. motionless position) every 1 s and % of time spent freezing was calculated [33].

### Histopathology

Immunohistochemical procedural methods were described by Gordon et al. [34]. For each marker, sections from all animals were placed in a multi-sample staining tray and endogenous peroxidase was blocked (10% methanol, 3% H_2_0_2_ in PBS; 30 min). Tissue samples were permeabilized (with 0.2% lysine, 1% Triton X-100 in PBS solution) and incubated overnight in appropriate primary antibody. Anti-NeuN (Millipore); anti-Aβ (prepared by Paul Gottschall), anti-GFAP (Dako), anti-Iba1 (Wako), total tau H150 (rabbit polyclonal, Santa Cruz Biotechnology), anti-pSer199/202 (rabbit polyclonal, Anaspec) and anti-pS396 tau (rabbit polyclonal, Anaspec) antibodies were used in this experiment. Sections were washed in PBS, and then incubated in corresponding biotinylated secondary antibody (Vector Laboratories, Burlingame, CA). The tissue was again washed after 2h and incubated with Vectastain® Elite® ABC kit (Vector Laboratories, Burlingame, CA) for enzyme conjugation. Finally, sections were stained using 0.05% diaminobenzidine and 0.03% H_2_0_2_. Tissue sections were mounted onto slides, dehydrated, and cover slipped.

Congo red and Gallyas histology were performed using sections that were premounted on slides and air-dried for a minimum of 24hours. The sections were rehydrated for 30 seconds before beginning staining protocol. For Congo red, 2.5mM NaOH was added to a saturated sodium chloride-ethanol solution and slides were incubated for 20 min. Subsequently, slides were incubated in 0.2% congo red in alkaline alcoholic saturated sodium chloride solution for 30 minutes. Slides were rinsed through three changes of 100% ethanol, cleared through three changes of xylene, and cover slipped with DPX. Gallyas staining was performed as described in [35]. Slides were treated with 5% periodic acid for 5 min, washed with water, and incubated sequentially in silver iodide (1 min) and 0.5% acetic acid (10 min) solutions prior to being placed in developer solution (2.5% sodium carbonate, 0.1% ammonium nitrate, 0.1% silver nitrate, 1% tungstosilicic acid, 0.7% formaldehyde). Slides were treated with 0.5% acetic acid to stop the reaction, then incubated with 0.1% gold chloride and placed in 1% sodium thiosulphate. Following a final wash in water, slides were dehydrated and cover slipped.

Stained sections were imaged using a Zeiss Mirax-150 digital scanning microscope and Image Analysis software. Area of positive stain for the entire brain region in each section (no sampling) was analyzed using 200x magnification. The software used hue, saturation and intensity (HSI) to segment the image fields. Thresholds for object segmentation were established with images of high and low levels of staining to identify positive staining over any background levels. These limits were held constant for the analysis of every section in each study [34].

### Statistics

Statistical analysis was performed using two-way ANOVA followed by Fisher’s LSD post hoc means comparison test using Stat View software version 5.0 (SAS Institute Inc, Cary NC). Graphs were generated using Graph Pad Prism 5.01 (La Jolla, CA).

## Results

### KD increased ketosis and reduced glucose levels

**Figure 2:**
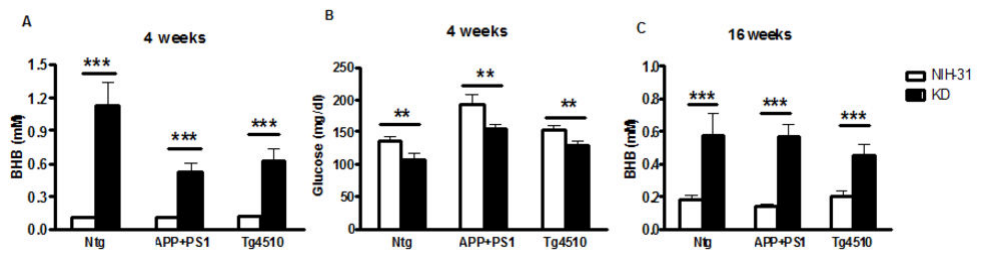
KD increased ketosis and reduced glucose levels. (A, C) KD (black bars) successfully increased peripheral β-hydroxybutyrate (BHB) levels after 4 weeks or 4 months, compared to a control diet (NIH-31, open bars). (B) Accordingly, circulating glucose levels were found to be decreased in KD-fed mice, in all genotypes. Glucose and BHB levels were measured utilizing a commercially available glucose/ketone monitoring system (Nova Max^©^ Plus). Data are presented as mean ± SEM (n=10). **p<0.0004, ***p<0.0001.

KD diet efficiently increased blood BHB levels when compared to NIH-31 control diet at 4 weeks (ANOVA main effect of diet, p<0.0001, Figure 2A). However, this increase was more pronounced in the nontransgenic genotype than in either APP+PS1 or Tg4510 transgenic lines (FLSD, p=0.013 and p=0.033, respectively), and there was a significant genotype and diet interaction (p=0.021). In addition, blood glucose levels were significantly decreased in KD-fed mice (ANOVA main effect of diet, p=0.002). A main effect of genotype was observed (p<0.0001) with APP+PS1 and Tg4510 mice presenting higher glucose levels compared to nontransgenic mice (FLSD, p<0.0001 and p=0.036, respectively; Figure 2B). Ketosis was shown to be maintained for the duration of the experiment, and blood BHB levels were also at higher levels in mice fed a KD at the 16 week time point (ANOVA main effect of diet, p<0.0001, Figure 2C).

### Genotype, but not diet, affected body weight, food intake and locomotor activity

**Figure 3:**
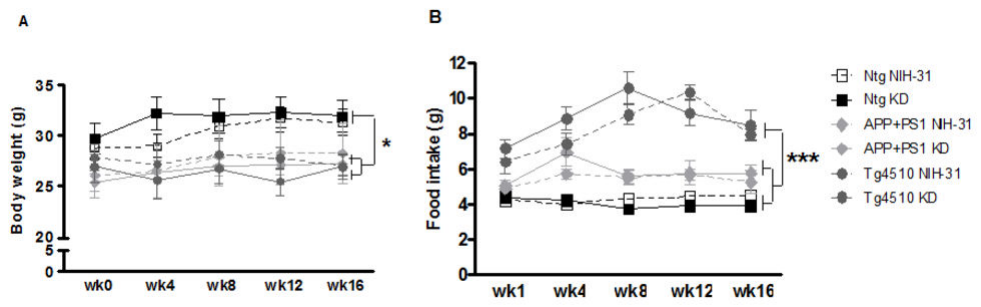
Changes in body weight and food intake throughout experiment. (A) Assessments of body weight and (B) food intake in APP+PS1, Tg4510 and nontransgenic mice on either control diet (NIH-31, open symbols) or ketogenic diet (KD, solid symbols) for 4 months. Both transgenic mouse lines weighed significantly less than nontransgenic mice. (B) Smaller body weights did not result from reductions in food intake. The Tg4510 mice ate significantly more food than did nontransgenic mice. Data are presented as mean ± SEM. *p<0.02, ***p<0.0001.

Average body weight was the same for all groups at the start of the experiment. An overall main effect of genotype on body weight was observed throughout the course of the study (ANOVA, p=0.018). At 16 weeks, both APP+PS1 and Tg4510 mice had smaller body weights, not affected by diet, relative to the nontransgenic mice (FLSD, p=0.018 and p=0.005, respectively; Figure 3A). Interestingly, the transgenic lines presented significantly greater food intake than nontransgenic control mice throughout the experiment, regardless of their diets (ANOVA main effect of genotype, p<0.0001, Figure 3B).

**Figure 4:**
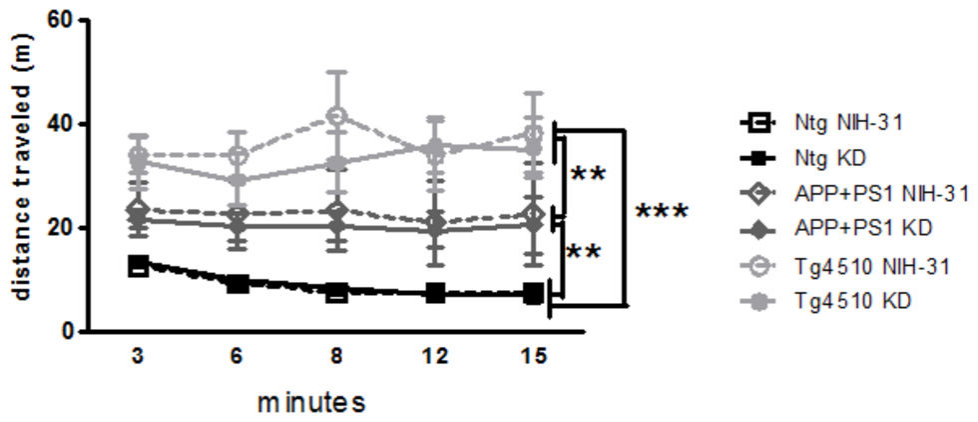
AD transgenic mouse models had increased locomotor activity in the open field. Both APP+PS1 and Tg4510 mice displayed greater total distance travelled in the open field, compared to nontransgenic control mice, regardless of the diet. Locomotor activity was assessed during a single 15min trial in the open field. Data are presented as mean ± SEM. **p<0.005 and ***p<0.0001.

Figure 4 shows that total distance travelled in the open field was significantly greater for both Tg4510 and APP+PS1 mice than for nontransgenic mice (ANOVA main effect of genotype, p<0.0001; FLSD, p<0.0001 and p=0.0043, respectively). Tg4510 showed even greater activity than APP+PS1 mice (FLSD: p=0.002), suggesting a possible hyperactive phenotype. In addition, Tg4510 mice made significantly more arm entries in the Y-maze test than nontransgenic controls (ANOVA main effect of genotype, p=0.012, Table 2). Furthermore, Tg4510 showed higher percentage of alternation in the Y-maze as well (ANOVA main effect of genotype, p=0.02, Table 2), when compared to APP+PS1 genotype (FLSD, p=0.009). No diet effects were observed in any genotype in either the open field or the Y-maze tests.

**Table 2:** Behavioral tests results.

Test	Ntg		APP+PS1		Tg4510	
	NIH-31	KD	NIH-31	KD	NIH-31	KD
Y-Maze						
Entries	42.4 ± 1.7	43.1 ± 2.0	49.7 ± 4.5	49.6 ± 5.8	67.8 ± 11.1*	50.2 ± 7.2*
% Alternation	62.9 ± 3.0	62.3 ± 3.1	51.6 ± 4.0	65.6 ± 4.8	72.0 ± 5.0^+^	70.8 ± 6.5^+^
Open Pool (s)	7.5 ± 1.1	8.3 ± 2.9	13.9 ± 0.7	18.3 ± 1.7	18.3 ± 1.6	14.3 ± 0.7
Context (% Freezing)	98.5 ± 0.8	94.3 ± 2.1	80.4 ± 9.1**	75.7 ± 7.2**	79.1 ± 7.7***	62.9 ± 8.3***

Data presented as mean ± SEM. FLSD, *p< 0.05, **p<0.006 and ***p=0.0002 compared to Ntg genotype. ^+^ p<0.01 compared to APP+PS1 genotype. Open pool results shown as seconds to reach a visible platform. Context results shown as % of freezing.

### KD enhanced motor performance but did not rescue memory deficits

**Figure 5:**
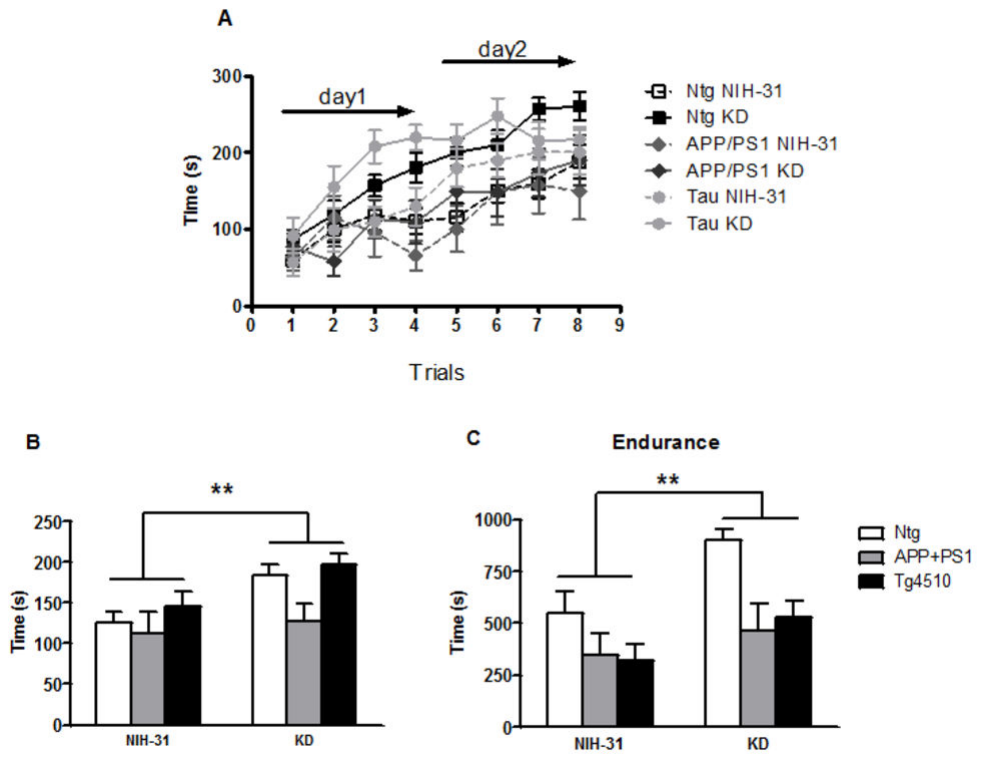
KD enhanced motor performance in all genotypes. Motor performance was assessed using two variations of the rotarod test. (A) Both genotype and diet effects were found in the standard accelerating rotarod test and (B) Average latency to fall in the accelerating rotarod was significantly greater in mice fed a KD. (C) Similarly, the ketogenic diet significantly enhanced motor performance in the non-accelerating variation of the test in all genotypes. Overall, latency to fall was greater in nontransgenic mice than in either APP+PS1 or Tg4510 mice. Data are presented as mean ± SEM. **p<0.007.

Mice fed a ketogenic diet spent significantly more time on the accelerating rotarod (Figure 5). Both genotype and diet affected their performance (ANOVA main effects, p=0.01 and p=0.005, respectively, Figure 5A). APP+PS1 mice performed poorly in comparison to nontransgenics (FLSD, p=0.05) or Tg4510 (FLSD, p=0.006). Figure 5B represents the averaged latency to fall for all trials (days 1 and 2) of the rotarod and highlights the KD-induced improvement in motor performance. In addition, in a variation of the rotarod paradigm where mice were tested for a maximum trial length of 1000 s while the rod was held constant at 25 rpm (lower maximum speed, Endurance trial), KD greatly enhanced motor activity and both genotype (p=0.002) and diet (p=0.006) main effects were observed (Figure 5C). Latency to fall in nontransgenic mice was significantly greater than APP+PS1 (FLSD, p=0.002) and Tg4510 (FLSD, p=0.003) mice (Figure 5B and 5C).

**Figure 6:**
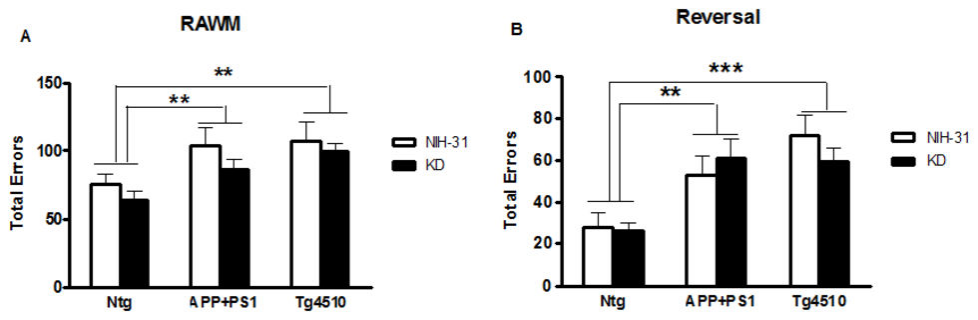
KD did not rescue spatial memory deficits. Spatial memory was assessed by the 2-day radial arm water maze (RAWM). (A) Both APP+PS1 and Tg4510 (tau) mice made more errors on the 2-day RAWM compared to Ntg control mice, regardless of the diet. (B) APP+PS1 and Tg4510 mice consistently made more entries into the wrong arms, suggesting that neither were able to learn a new platform location on the reversal trial. Data are presented as mean ± SEM. **p<0.01 and ***p<0.0001.

Spatial memory deficits were assessed by the 2-day radial arm water maze. ANOVA main effect of genotype (p=0.001) showed that both APP+PS1 and Tg4510 made significantly more errors in the attempt to find the platform compared to nontransgenic control groups (FLSD, p=0.01 and p<0.001, respectively), regardless of the diet (Figure 6A). Neither transgenic line performed as well on the reversal trial as the nontransgenic controls (ANOVA main effect of genotype, p<0.0001; FLSD APP+PS1, p<0.001 and Tg4510, p<0.0001, compared to Ntg). The open pool test showed that all mice were capable of performing a visual platform test (Table 2).

Diet did not affect the freezing response during the training session of the CFC (data not shown). Memory for contextual fear tested 24hours after training session was not affected by diet but a genotype effect was observed (p=0.0004) with APP+PS1 and Tg4510 mice freezing significantly less than the nontransgenic groups (Table 2; FLSD, p=0.005 and p=0.0002, respectively).

### Neuronal loss in Tg4510 mice was not rescued by KD and was associated with increased astrocytosis and microglial activation

**Figure 7:**
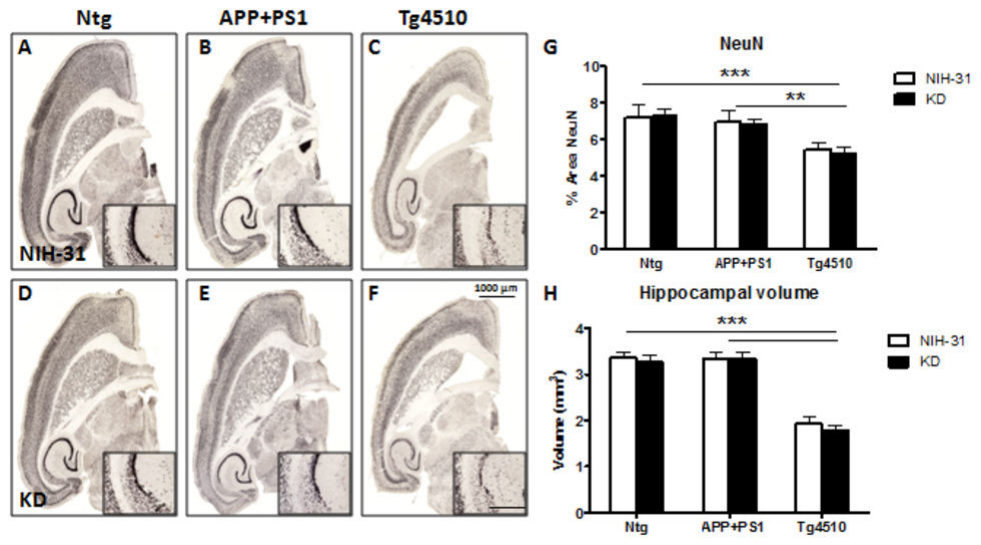
KD did not rescue neuronal loss in Tg4510 mice. Micrograph representation (2X) of neuronal staining (NeuN) in Ntg (A, D), APP+PS1 (B, E) and Tg4510 (C, F) mice kept on either NIH-31 (A, B, C) or KD diets (D, E, F). (G) Immunoreactivity for the neuronal marker NeuN was significantly reduced in Tg4510 mice compared to both nontransgenic and APP+PS1 mice. (H) Hippocampal volume (expressed in mm^3^) was calculated in Nissl stained sections. In agreement with the neuronal loss observed, hippocampal volume was significantly smaller in Tg4510 line, compared to the other genotypes. No diet effects were observed. Immunostaining was digitally quantified by Mirax image analysis. Data are presented as mean ± SEM. 2X Scale bar = 1000μm; 20X inset scale bar = 100 μm. **p<0.01 and ***p<0.0001.

Tg4510 mice overexpress human tau with a P301L mutation and have been reported to exhibit significant neuronal loss by 6 months of age [30]. Mice in this experiment were 9 months old at the time of euthanasia. To investigate whether our dietary manipulation affected total neuronal loss in our mouse lines, representative sections were stained with a biotinylated antibody against neuron-specific protein NeuN. Indeed, we found estimated reductions of 23% and 27% in area immunoreactive for NeuN in the brains of Tg4510 mice, when compared to APP+PS1 or nontransgenic mice, respectively (FLSD, p=0.001 and p<0.0001, Figure 7G). This difference, indicative of the significant neuronal loss observed in this model, was not prevented by a KD. As expected, APP+PS1 mice did not show any differences in the percentage of NeuN positive cells when compared to nontransgenic groups, regardless of the diet. Accordingly, the hippocampal volume (expressed in mm^3^) of Tg4510 mice was significantly smaller than either APP+PS1 or nontransgenic genotypes (FLSD, p<0.0001).

**Figure 8:**
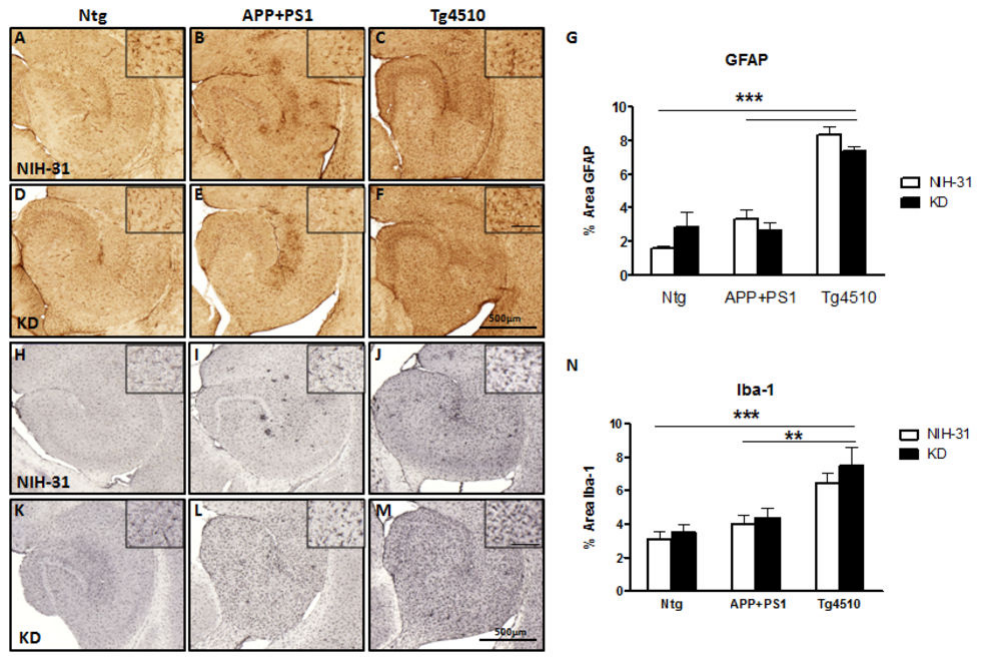
Astrocytosis and microglial activation were observed in Tg4510 mice. Micrograph representation (5X) of hippocampi stained for GFAP positive astrocytes (panels A-G) or Iba-1 positive microglia (panels H-N) in Ntg (A, D, H, K), APP+PS1 (B, E, I, L) and Tg4510 (C, F, J, M) mice kept on either NIH-31 (A, B, C, H, I, J) or KD diets (D, E, F, K, L, M). (G, N) Immunostaining was digitally quantified by Mirax image analysis. Data are presented as mean ± SEM. Scale bar = 500μm; inset scale bar = 100 μm ***p<0.0001.

In addition, Tg4510 mice showed prominent GFAP-positive reactive astrocytes in comparison to either age-matched nontransgenic controls or APP+PS1 mice (FLSD, p<0.0001), Figure 8 (panels A-G). Microglial activation was similarly elevated in the Tg4510 transgenic mice, compared to nontransgenic controls or APP+PS1 mice (FLSD, p<0.0001 and p=0.001, respectively), as shown in Figure 8 (panels H-N). These changes are consistent with the presence of degenerating neurons No diet effects were observed for either astrocyte or microglial markers.

### No changes in amyloid or tau deposition were observed

Results from histological assessments are detailed in Table 3. As expected, no Aβ or tau immunoreactivity was observed in nontransgenic control mice, regardless of diet.

**Table 3:** Immunohistochemical markers.

	APP+PS1		Tg4510	
Markers (% Area)	NIH-31	KD	NIH-31	KD
Abeta	2.4 1± 0.89	2.83 ± 0.68	nd	nd
Congo	0.23 ± 0.02	0.25 ± 0.01	nd	nd
H150	nd	nd	0.46 ± 0.04	0.49 ± 0.07
Ser 199/202	nd	nd	0.95 ± 0.15	0.99 ± 0.11
Ser 396	nd	nd	0.76 ± 0.10	0.71 ± 0.06
Gallyas	nd	nd	2.01 ± 0.23	1.65 ± 0.16

% Positive Area of staining shown. Data presented as mean ± SEM. nd: not detected

Whole sections were analyzed and no changes in Aβ immunoreactivity or congophilic compact plaques were observed in APP+PS1 mice fed a ketogenic diet for 4 months. Additionally, no changes in the percentage of area immunoreactive for total tau or phosphorylated tau were observed in Tg4510 mice on the ketogenic diet, compared to the control diet. Total tau immunoreactivity was assessed by staining with H150 antibody (against aa 1-150), which recognizes both human and mouse tau. Antibodies against tau phosphorylated at epitopes Ser 199/202 and Ser 396 were also used in this experiment. Moreover, Gallyas silver staining against deposited fibrillar tau was also performed. KD did not change any of the tau pathology markers used in our study. Moreover, cortical and hippocampal brain regions were analyzed separately and no differences were found between diets for either tau or amyloid deposition in these selected areas (data not shown).

## Discussion

In our experiment, we were able to effectively induce ketosis reaching 1 mM ketone body levels adult transgenic and nontransgenic mice with a specially designed MCT-rich, low carbohydrate ketogenic diet. Accordingly, plasma glucose levels were significantly diminished in KD-fed mice. Four months later, at the end of the study, plasma BHB levels were still increased when compared to mice fed a standard, NIH-31 diet (Figure 2). Therefore, we showed that our diet was successful in inducing and maintaining therapeutic ketosis throughout the course of the experiment.

Many previous reports associate KDs with weight loss in both humans and rodents [36-42]. Additionally, KDs are often associated with low palatability and, therefore, voluntary caloric restriction [43]. Previously we demonstrated that caloric restriction slows amyloid deposition in APP+PS1 mice [44]. However, in our experiment, KD-fed mice did not reduce food intake or display decreased body weight (Figure 3). Therefore, caloric restriction was not a confound in this experiment. The failure of the ketogenic diet to slow amyloid deposition would imply that the ketosis associated with caloric restriction is unlikely to mediate the effects of caloric restriction on the APP+PS1 mouse phenotype.

Genotype, but not diet, was found to affect body weight, with transgenic mouse lines presenting smaller body weights. However, most surprising in this regard was the significantly greater food intake than nontransgenic age-matched controls. One possible explanation for this could be increased locomotor activity associated with the Tg4510 mouse line, and to a lesser extent with the APP+PS1 mice, in both the open field (Figure 4) and y-maze arm entries (table 2). It is also feasible that the Tg4510 mice have a difference in basal metabolism. In agreement with this observation, Morris et al. [45] found that the ablation of tau (Tau^-/-^) caused weight gain in middle-aged mice. This is suggestive of tau playing a possible role in the hypermetabolic phenotype encountered in this study. Indeed, hypermetabolism in amyloid models has been previously reported [46,47]. Anecdotally, it has been reported that dementia patients sometimes exhibit significant weight loss, associated with increased intake of calories per kg body weight [48]. We are planning calorimetry studies in the future to evaluate these options more completely.

In our study, KD-fed mice presented significantly enhanced motor performance, regardless of genotype (figure 5). Our findings suggest that, despite the fact that all genotypes showed motor learning after one day of training (figure 5A), mice that were fed a low carbohydrate diet presented significantly greater latency to fall from the rotarod in both the high and low maximum rotation speed versions of the task (figure 5B and C, respectively). There is a possibility that the enhanced performance observed could be due to motor learning, which permitted them to stay longer on the rod. Alternatively, we suggest that this improved motor performance could be due to the KD-induced increase in metabolic efficiency in muscles. Several recent studies reported the beneficial effects of KDs on motor performance in different mouse models. A medium chain triglyceride diet improved rotarod performance in a mouse model of amyotrophic lateral sclerosis (ALS) [18], despite the lack of an increase in survival. The authors attributed this effect to the preservation of motor neurons in the spinal cord at the end stage of the disease. This enhanced motor performance was recently shown to be duplicated by supplementing typical rodent diets with 10% caprylic acid, a medium chain triglyceride that elevated plasma ketones to 0.5 mM. Using a mouse model of experimental autoimmune encephalomyelitis (EAE), Kim et al [21] showed that feeding a KD diet starting 7d before induction diminished all components of the EAE phenotype. In addition to improving motor disability scores, KD-fed EAE mice showed significantly shorter latencies to find the platform in a water maze. This effect possibly resulted from their increased swim speed, compared to EAE mice fed a control diet. Rescue of deficits in long term potentiation, reduced lesion volumes, fewer infiltrating T cells and reduced inflammatory cytokine levels were reported in mice offered KD. In humans, Paoli et al [38] demonstrated that artistic gymnasts on a very low carbohydrate KD for 30 days decreased body weight and fat without negative effects on strength performance in athletes.

In agreement with our results, a recent study reported that KD improved rotarod performance in both nontransgenic and APP+PS1 mice when fed KD for one month [49]. This diet also elevated ketones to 1 mM, as in the present study. The mice in this study were quite young (1-2 mo) and not yet depositing amyloid in the brain, but no effects on the soluble amyloid in brain or muscle could be detected by ELISA, consistent with the present results. Another recent study [36] described effects of supplementing the diets of 3xTg mice with a ketone ester, providing 20% of calories and reaching 0.7 mM BHB. These mice were treated for 4-7 mo and the authors observed subtle differences in learning, decreased anxiety and increased locomotor activity in the ketone- supplemented mice. Unfortunately, no nontransgenic mice were included to aide in determining if these differences represented a) a worsening or rescue of the transgenic phenotype or b) a general effect observed in all mice or a specific interaction with the transgenic mouse phenotype. Although these mice did not deposit amyloid, they did exhibit intracellular staining with an anti-Aβ antiserum. The number of cells exhibiting this staining was reduced by the ketone ester supplement. This contrasts with our inability to discern improvements in spatial or associative memory deficits or reductions in amyloid deposits in APP+PS1 mice offered the KD.

KD did not rescue neither global neuronal loss nor hippocampal atrophy in Tg4510 mice. These are hallmarks of AD and are replicated in the Tg4510 mouse model, with widespread development of neurons bearing hyperphosphorylated tau and later cell death [29,30,50,51]. Indeed, we did observe a significant decrease in the fractional area occupied by NeuN staining in the Tg4510 genotype, but the ketogenic diet did not prevent it. It is possible that small differences in select neuronal populations were not detected using this analysis. We did not observe a decrease in NeuN staining in the APP+PS1 genotype, an amyloid overexpression model. As previously observed [50], neuronal loss in Tg4510 mice was accompanied by an increase in GFAP-positive reactive astrocytes and microglial activation [25] (Figure 8). Markers for tau pathologies were also analyzed in Tg4510 mice. Significant numbers of neurons were positive for tau, phosphorylated tau epitopes and argyophilic tau, but no differences in the amount of staining were observed after 4 months of KD treatment (Table 3).

Yao et al [40] reported that treatment with 2-deoxyglucose significantly reduced both mitochondrial APP and the 16kD Abeta oligomer levels. Because 2-DG competitively blocks glucose metabolism, it induces a compensatory rise in alternative substrates, primarily ketone bodies by the liver, suggesting that other mechanisms activated by CR may underlie its neuroprotective effects observed in reducing amyloid pathology [44]. Despite significant reductions in amyloid markers in their study, tau hyperphosphorylation remained unchanged, suggesting that different mechanisms are possibly at play in tau pathology. This lack of effect on tau pathology contrasts with the finding using ketone ester supplementation in 3xTg mice [36], where treatment decreased the number of cells staining for PHF-tau by roughly one third.

In summary, our results suggest that a MCT-rich, low carbohydrate ketogenic diet, significantly induced ketosis and improved motor performance in all genotypes tested in our study. In the past few years, increasingly anecdotal evidence of the benefits of KD has been reported in AD or MCI patients. Clinical trials involving Parkinson’s and Alzheimer’s disease patients treated with diet-induced hyperketonemia resulted in improved motor function [22] and enhanced cognition [23], respectively. These benefits tend to appear in a relatively short time frame (weeks to several months). Our data testing the hypothesis that these dietary manipulations would impact the deposition of amyloid or tau and slow the underlying disease process is not supported by the data we obtained in this study. Instead, the behavioral improvements observed are likely due to a metabolic effect, enhancing the performance of remaining neurons, while the underlying disease proceeds unabated. Therefore, KD may play an important role in improving motor performance and providing symptomatic relief to individuals with Alzheimer’s and/or other dementias, but would seem unlikely to modify the rate of accumulation of the neuropathology in these disorders.
